# Protective effects of hydrogen-rich saline on monocrotaline-induced pulmonary hypertension in a rat model

**DOI:** 10.1186/1465-9921-12-26

**Published:** 2011-03-04

**Authors:** Yun Wang, Lei Jing, Xiao-Min Zhao, Ji-Ju Han, Zuo-Li Xia, Shu-Cun Qin, Ya-Ping Wu, Xue-Jun Sun

**Affiliations:** 1 Artherosclerosis Research Institute of Taishan Medical University, Taian 271000, P.R.China; 2 Province Key Laboratory of Oral and Maxillofacial, Head and Neck Medical Biology Laboratory, Liaocheng People's Hospital, Taishan Medical University, Liaocheng252000, P.R.China; 3 Department of Clinical Chemistry and Haematology, University Medical Center Utrecht, PO Box 85500, 3508 GA Utrecht, The Netherlands; 4 Department of Diving Medicine, the Second Military Medical University, Shanghai 200433, P. R. China

## Abstract

**Background:**

Hydrogen-rich saline has been reported to have antioxidant and anti-inflammatory effects and effectively protect against organ damage. Oxidative stress and inflammation contribute to the pathogenesis and/or development of pulmonary hypertension. In this study, we investigated the effects of hydrogen-rich saline on the prevention of pulmonary hypertension induced by monocrotaline in a rat model.

**Methods:**

In male Sprague-Dawley rats, pulmonary hypertension was induced by subcutaneous administration of monocrotaline at a concentration of 6 mg/100 g body weight. Hydrogen-rich saline (5 ml/kg) or saline was administred intraperitoneally once daily for 2 or 3 weeks. Severity of pulmonary hypertension was assessed by hemodynamic index and histologic analysis. Malondialdehyde and 8-hydroxy-desoxyguanosine level, and superoxide dismutase activity were measured in the lung tissue and serum. Levels of pro-inflammatory cytokines (tumor necrosis factor-α, interleukin-6) in serum were determined with enzyme-linked immunosorbent assay.

**Results:**

Hydrogen-rich saline treatment improved hemodynamics and reversed right ventricular hypertrophy. It also decreased malondialdehyde and 8-hydroxy-desoxyguanosine levels, and increased superoxide dismutase activity in the lung tissue and serum, accompanied by a decrease in pro-inflammatory cytokines.

**Conclusions:**

These results suggest that hydrogen-rich saline ameliorates the progression of pulmonary hypertension induced by monocrotaline in rats, which may be associated with its antioxidant and anti-inflammatory effects.

## Background

Pulmonary hypertension (PH), a syndrome that encompasses several diseases, is characterized by a progressive elevation of pulmonary arterial pressure, which may ultimately induce right ventricular (RV) failure and death [1]. Pulmonary hypertension, either idiopathic or secondary, may share some of the following pathological or functional changes, including vascular remodeling, endothelial dysfunction/increased vasoconstriction, oxidative stress and inflammation. Among these changes, the effects of oxidative stress and inflammation on PH have been investigated intensively in recent years. Oxidative stress is characterized by an increase in oxidants with or without a decrease in antioxidants or antioxidant enzymes. Oxidants cause tissue damage by mechanisms such as lipid peroxidation and DNA damage [2]. Previous studies have suggested that increased oxidative stress contributes to the pathogenesis and/or development of PH [3], and that antioxidant treatment ameliorates PH or PH-induced heart failure in rats [4,5]. Furthermore, the mechanisms of inflammation in PH include up-regulation of cytokines and infiltration of inflammatory cells.

Current treatment for PH is limited and only provides symptomatic relief. Therefore, it is imperative to look for new therapeutic approach for PH. Hydrogen gas (H_2_) has been applied in medical applications to prevent decompression sickness [6]. Shirahata and colleagues [7] reported that electrolyzed-reduced water, which dissolved large amounts of H_2_, had the ability to protect DNA from oxidative damage. Recently, it has been suggested that H_2 _has therapeutic antioxidant activity by selectively reducing hydroxyl radicals and effectively protecting against organ damage, such as cerebral ischemia, neonatal cerebral hypoxia-ischemia, liver injury, lung injury and myocardial injury induced by ischemia/reperfusion [8-12]. Moreover, it has been reported that hydrogen-rich saline has an anti-inflammatory effect [13].

Therefore, we hypothesized that the antioxidant and anti-inflammatory effects of hydrogen-rich saline might prevent the progression of PH. To test this hypothesis, we investigated the efficacy of hydrogen-rich saline in monocrotaline (MCT)-treated PH rats.

## Methods

### Animals

Male Sprague-Dawley rats, weighing 200-220 g, were provided by the Experimental Animal Center of Shandong University of Traditional Chinese Medicine (Shandong, China). Rats were housed with free access to food and water under a natural day/night cycle. Rats were acclimated for 7 days before any experimental procedures. All rats received humane care according to the Guide for the Care and Use of Laboratory Animals by the Chinese Academy of Sciences.

### Drugs and materials

Hydrogen-rich saline was prepared as previously described [14]. Briefly, hydrogen was dissolved in normal saline for 2 h under high pressure (0.4 MPa) to the supersaturated level using a self-designed, hydrogen-rich water-producing apparatus. The saturated hydrogen-saline (250 ml) was stored under atmospheric pressure at 4°C in an aluminum bag without dead volume. Hydrogen-rich saline was freshly prepared every week to ensure a constant concentration of greater than 0.6 mM. Monocrotaline was purchased from Wako Pure Chemical Industries, Ltd.(Osaka Japan). Malondialdehyde (MDA) and superoxide dismutase (SOD) assay reagents were obtained from Nanjing Jiancheng Bioengineering Institute (Nanjing, China). Tumor necrosis factor-α (TNF-α), interleukin-6 (IL-6) and 8-hydroxy-desoxyguanosine (8-OHdG) Enzyme-Linked Immunosorbent Assay (ELISA) kits were purchased from Shanghai Bluegene Biotech Co., Ltd. (Shanghai, China).

### Experimental design

Rats were divided randomly into the following groups of 10 rats each: (1) control group, in which rats received an equal volume of vehicle, followed by saline from day 1 to day 21; (2) MCT-treated group, in which rats received a single subcutaneous injection of MCT (dissolved in 1N HCL buffered to pH 7.0 with 1N NaOH [15]) at a dose of 6 mg/100 g body weight, followed by saline from day 1 to day 21; (3) hydrogen-rich saline 2-week group, in which rats received hydrogen-rich saline from day 8 to day 21 after MCT injection; (4) hydrogen-rich saline 3-week group, in which rats received hydrogen-rich saline from day 1 to day 21 after MCT injection. Either 5 ml/kg hydrogen-rich saline or the same volume of vehicle (saline) was administrated once daily by intraperitoneal (i.p.) injection. All the experiments were approved by the Animal Care Ethics Committee of Taishan Medical University (Taian China).

### Hemodynamic studies

On day 22, rats were anesthetized with 10% chloral hydrate (0.4 ml/100 g body weight, i.p.) and placed in a supine position. According to Sun's method [16], MP150 system (BIOPAC, USA) was applied in our experiments. Briefly, a polyethylene catheter was introduced into the right ventricle through the jugular vein to measure right ventricular systolic pressure (RVSP). Peak rates of RV pressure rise (dP/dt max) and pressure fall (dP/dt min) were measured as well. The catheter was advanced to the pulmonary artery to measure mean pulmonary artery pressure (mPAP). After hemodynamic measurements, the thorax was opened, blood was taken from the heart for serum preparation, and lung and heart were processed for histological evaluation or frozen in liquid nitrogen for further analysis.

### Measurement of RV hypertrophy[17]

Heart was dissected and weighed, and the ratio of RV weight to left ventricle plus septum weight (RV/[LV+S] weight) was measured and calculated.

### Histopathological observations

For histopathological observations, specimens of the right lower lung were harvested and flushed with normal saline, fixed in 4% paraformaldehyde for 24 h, and embedded in paraffin. Sections of 4 μm were stained with hematoxylin-eosin (H-E) for light microscopy.

### Determination of TNF-α and IL-6 levels in the serum

Levels of TNF-α and IL-6 in serum were measured with commercial ELISA kits following the instructions of the manufacturer. Absorbance was read on a microplate reader and the concentrations were calculated according to the standard curve.

### Measurement of 8-OHdG, MDA and SOD in lung tissues and serum

Left lung tissues (100 mg, wet wt.) were homogenized in 1 ml saline at 4°C. The homogenates were centrifuged at 2000 rpm at 4°C for 15 min. The MDA content and SOD activity in both supernatant and serum were determined by chemical assay according to the manufacturer's instructions. Levels of 8-OHdG in serum and lung tissue were measured with ELISA kits. Protein concentration was measured using the Bradford method, and the results were expressed as microgram of protein.

### Statistics

Results were expressed as mean ± S.D. All data were statistically analyzed with SPSS11.5 (SPSS Inc., Chicago, IL, USA). Statistical comparisons were performed by one-way analysis of variance (ANOVA) followed by Student-Newman-Keuls's post hoc test. A *P *value less than 0.05 was considered statistically significant.

## Results

### Hydrogen-rich saline treatment improved hemodynamics

**Figure 1:**
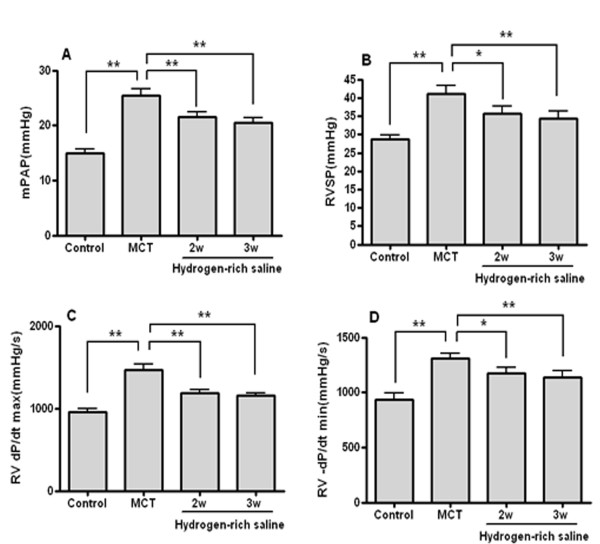
**Hydrogen-rich saline improved hemodynamics in MCT-induced PH**. mPAP (A), RVSP (B), RV dP/dt max (C) and RV -dP/dt min (D). *P < 0.05, **P < 0.01.

Results of hemodynamic studies in the four groups are shown in Figure 1. Compared with the control group, mPAP, RVSP, RV dP/dt max and dP/dt min in rats challenged with MCT in the MCT-treated group increased significantly (*P *< 0.01), indicating that rats developed severe PH. Hydrogen-rich saline treatment for either 2 or 3 weeks attenuated the effects of MCT, suggesting that mPAP, RVSP, RV dP/dt max and dP/dt min were decreased significantly compared with the MCT group (*P *< 0.05).

### Hydrogen-rich saline treatment ameliorated the damage to lung tissue and reversed RV hypertrophy

**Figure 2:**
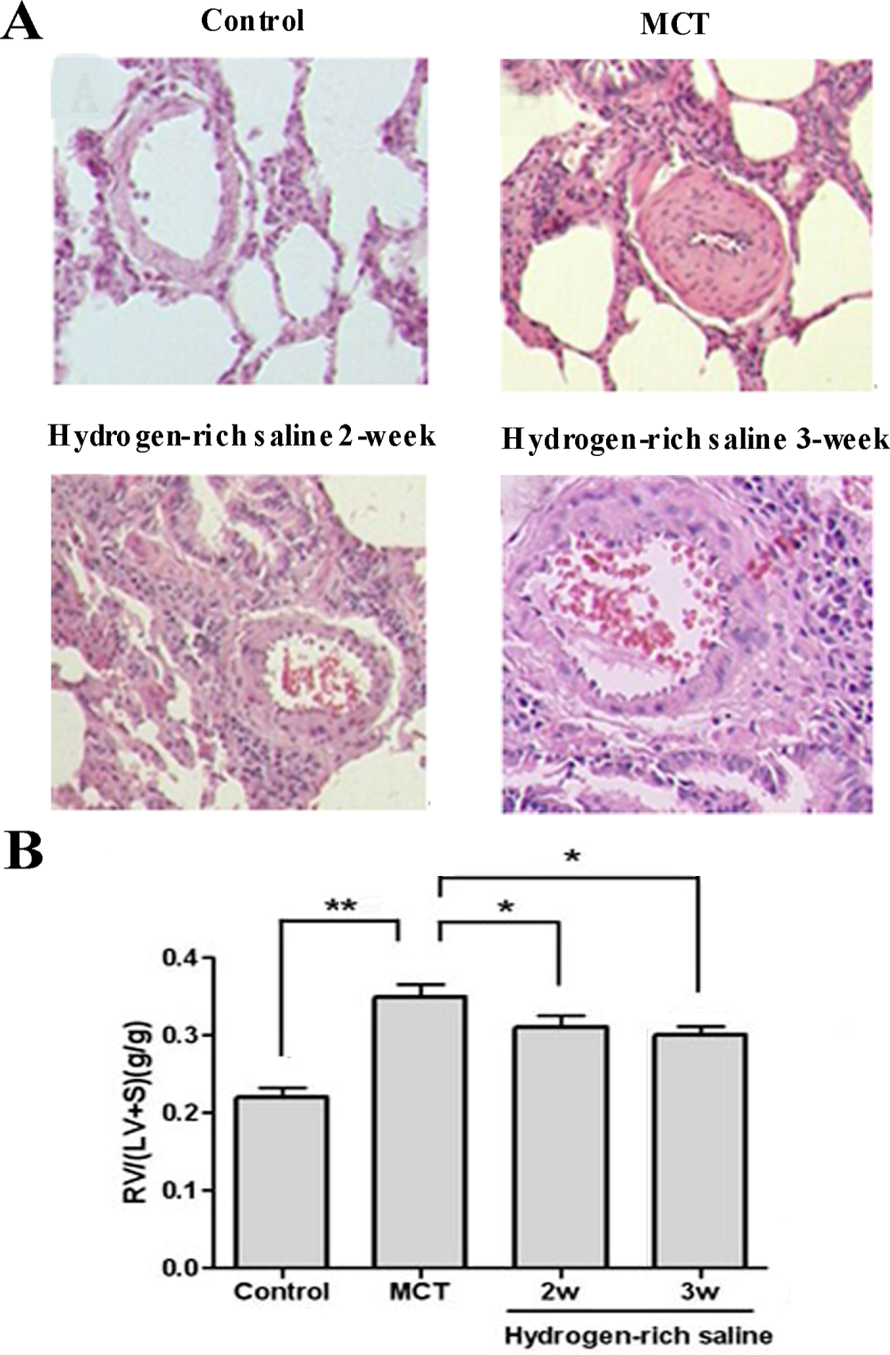
**Representative photomicrographs of right lower lung sections and RV hypertrophy index**. Lung sections in the control group showed normal architecture. Lung sections from the MCT-treated group showed tissue damage characterized by a thicker pulmonary artery wall, lumen stenosis, and inflammatory cell infiltration. Lung sections from rats treated with hydrogen-rich saline (5 ml/kg once daily for 2 or 3 weeks) showed significantly less histological alteration. Sections were stained with H-E (200×) (A). Administration of hydrogen-rich saline significantly reduced RV hypertrophy compared to the MCT-treated group (B). *P < 0.05, **P < 0.01.

In the lungs of MCT-treated rats, the pulmonary artery wall was significantly thicker, the medial smooth muscle layer was increased significantly, and the lumen appeared stenosed or occluded. Large amounts of inflammatory cells infiltrated the lung tissue. However, all of these pathological changes were decreased by the hydrogen-rich saline treatment (Figure 2A).

With regard to RV hypertrophy, the ratio of RV weight to LV+S weights in the MCT group (0.35 ± 0.04, *P *< 0.01 versus the control group) increased significantly compared with the control group (0.22 ± 0.03), indicating that RV hypertrophy developed as a consequence of increased pulmonary pressure. After 2 or 3 weeks of hydrogen-rich saline treatment, the ratio of RV weight to LV+S weights fell significantly to 0.31 ± 0.04 (*P *< 0.05 versus the MCT group) and 0.30 ± 0.03 (*P *< 0.05 versus the MCT group). These data showed that hydrogen-rich saline could reverse MCT-induced RV hypertrophy (Figure 2B).

### Hydrogen-rich saline treatment reduced the TNF-α and IL-6 levels in serum

**Figure 3:**
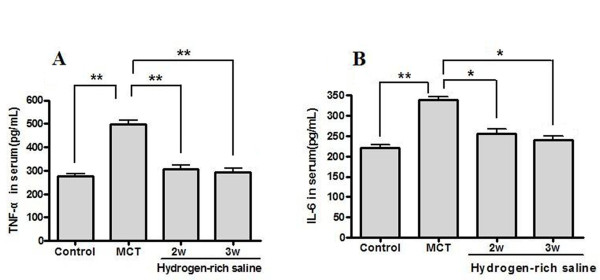
**Effects of hydrogen-rich saline treatment on serum levels of TNF-α and IL-6**. Administration of hydrogen-rich saline (5 ml/kg once daily for 2 or 3 weeks) significantly reduced the elevation of TNF-α (A) and IL-6 (B) in MCT-induced PH. *P < 0.05, **P < 0.01.

ELISA detection showed that the levels of TNF-α and IL-6 in the serum were markedly increased in the MCT group (496.21 ± 53.73 pg/ml and 339.38 ± 20.75 pg/ml, respectively) compared with the control group (275.65 ± 32.31 pg/ml and 220.13 ± 25.01 pg/ml, respectively). Hydrogen-rich saline treatment for 2 weeks (305.85 ± 50.49 pg/ml and 255.11 ± 34.59 pg/ml, respectively) or 3 weeks (293.17 ± 51.26 pg/ml and 241.00 ± 23.43 pg/ml, respectively) reduced the elevation of TNF-α and IL-6 (Figure 3).

### Hydrogen-rich saline treatment decreased MDA and 8-OHdG concentrations and increased SOD activity in serum and lung tissues

**Figure 4:**
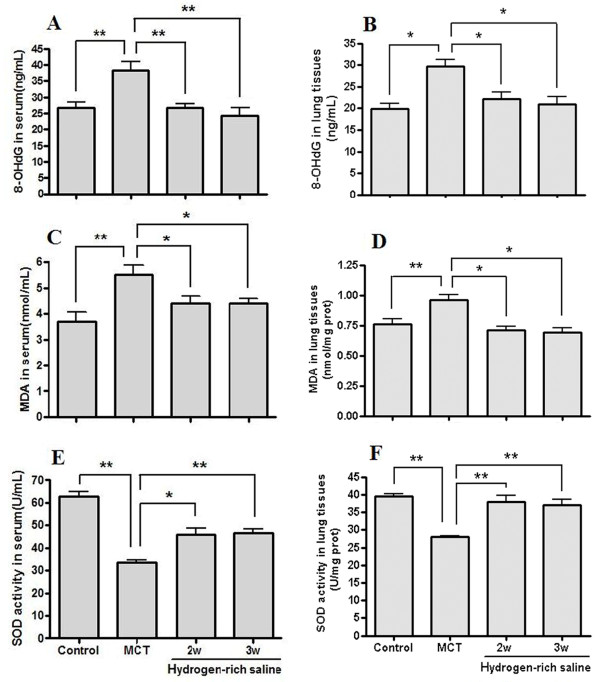
**Changes in 8-OHdG and MDA levels, and SOD activity in serum and lung tissue**. Hydrogen-rich saline treatment (5 ml/kg once daily for 2 or 3 weeks) significantly decreased the 8-OHdG (A and B) and MDA (C and D) levels and increased SOD (E and F) activity in serum and lung tissues. *P < 0.05, **P < 0.01.

Concentrations of MDA and 8-OHdG in serum and lung tissue from the MCT group were higher and SOD activity was lower than in control group. It was noted that hydrogen-rich saline treatment for either 3 or 2 weeks significantly decreased the MDA and 8-OHdG levels and increased SOD activity compared with the MCT group (Figure 4).

## Discussion

This study demonstrated that hydrogen-rich saline treatment could prevent the development of PH and reverse RV hypertrophy induced by MCT in a rat model. This observation was supported by the results from hemodynamic studies and histological findings. In addition, hydrogen-rich saline decreased MDA and 8-OHdG levels and increased SOD activity in lung tissue and serum, accompanied by a reduction of various cytokines (TNF-α, IL-6).

Monocrotaline, a pyrrolizidine alkaloid, has no intrinsic activity. In the liver, it is transformed by monooxygenase to bioactive monocrotaline pyrrole, which selectively injures the vascular endothelium of lung vessels. Progressive pulmonary vasculitis leads to increased vascular resistance and a gradual increase in arterial pressure beginning approximately 7 days after a single dose of MCT [18]. In our study, the rat model mimics several aspects of both primary and secondary human PH, including vascular remodeling, proliferation of pulmonary arterial smooth muscle cells, oxidative stress, endothelial dysfunction, upregulation of inflammatory cytokines, and leukocyte infiltration [19]. A group treated with hydrogen-rich saline one week after MCT administration was included in our study, in order to avoid having the antioxidant activity of hydrogen-rich saline interfere with the transformation of MCT in the liver. Based on the results, we can presume that hydrogen-rich saline had no effect on this process. Furthermore, we have also measured the hemodynamic and RV hypertrophy index of rats at one week after MCT administration with or without giving hydrogen-rich saline, and found that only mPAP increased slightly compared with control rats and hydrogen-rich saline had no effect in just one week (data not shown). So we selected three weeks after MCT administration as the end-point of our experiment.

Previous studies have focused on the effects of hydrogen-rich saline on organ damage been induced by ischemia/reperfusion. However, the effect of hydrogen-rich saline on PH remains unclear. In our study, prevention of progression of PH was observed with hydrogen-rich saline therapy, which also reduced adaptive hypertrophy of the right ventricle. Structural changes observed in MCT-induced pulmonary hypertension also were attenuated by hydrogen-rich saline treatment, as shown in our histopathological study. Current research indicates that inflammation contributes to the development of PH [20]. In our animal model of PH, the amount and activity of several inflammatory cells were increased, including macrophages, and neutrophils. TNF-α and IL-6, the signaling molecules, were released from activated macrophages and neutrophils, and exhibited an amplifying effect on the inflammatory response. Serum TNF-α and IL-6 levels were upregulated significantly in the MCT-treated group, while the serum TNF-α and IL-6 levels were down-regulated significantly by treatment with hydrogen-rich saline. These results suggest that the effects of hydrogen-rich saline on PH might be mediated by depression of TNF-α and IL-6, and that hydrogen-rich saline also has anti-inflammatory activity.

There is solid evidence that oxidative injury to the pulmonary vascular endothelium in MCT-treated rats precedes the progression of PH [3,21]. 8-Hydroxy-deoxyguanosine (8-OHdG) is a product of DNA oxidative damage caused by reactive oxygen species, and the level can not be influenced by diet or cell renewal. Therefore, 8-OHdG might be a new biomarker to assess DNA oxidative damage and oxidative stress [22]. Malondialdehyde is the ultimate product of unsaturated lipid peroxidation. The measurement of malondialdehyde in the blood may provide information on an excessive generation of free radical-induced membrane injury. Superoxide dismutase, an important antioxidant enzyme in the regulation of oxidative tissue damage, may catalyze the dismutation of two superoxide radicals to hydrogen peroxide and oxygen. In this study, we found that 8-OHdG and MDA levels were increased and SOD activity was decreased in lung tissue and serum in the MCT-treated group compared to the control group. In contrast, hydrogen-rich saline treatment significantly decreased the 8-OHdG and MDA content and increased SOD activity, consistent with its anti-oxidative effect.

## Conclusions

This study shows that hydrogen-rich saline treatment ameliorates the progression of PH induced by MCT in rats, which may be associated with its anti-inflammatory and antioxidant effects. Our findings suggest that hydrogen-rich saline may be beneficial for the treatment of PH. Future studies are needed to examine (1) the effects of hydrogen-rich saline is preventive, therapeutic, or both and time-course analysis would be needed and (2) the detailed molecular mechanism of hydrogen-rich saline on PH.

## Abbreviations

8-OHdG: 8-hydroxy-deoxyguanosine; dP/dt max: peak rates of RV pressure rise; dP/dt min: peak rates of RV pressure fall; ELISA: Enzyme-Linked Immunosorbent Assay; H-E: hematoxylin-eosin;IL-6: interleukin-6; MCT: monocrotaline; MDA: malondialdehyde; mPAP: mean pulmonary artery pressure; PH: pulmonary hypertension; RV: right ventricular; RVSP: right ventricular systolic pressure; SOD: superoxide dismutase; TNF-α:tumor necrosis factor-α.

## Competing interests

The authors declare that they have no competing interests.

## Authors' contributions

YW, LJ and YPW carried out rat experiments and immunoassays, performed histological analyses, and helped to draft the manuscript. XJS and XMZ conceived and designed and coordinated the study, analyzed the data, and wrote the manuscript. JJH performed the analyses and participated in data acquisition. ZLX and SCQ participated in the design and provided expert consultation. All authors read and approved the final manuscript.
